# BBMerge – Accurate paired shotgun read merging via overlap

**DOI:** 10.1371/journal.pone.0185056

**Published:** 2017-10-26

**Authors:** Brian Bushnell, Jonathan Rood, Esther Singer

**Affiliations:** 1 DOE Joint Genome Institute, Walnut Creek, CA, United States of America; 2 National Renewable Energy Laboratory, Golden, CO, United States of America; Massey University, NEW ZEALAND

## Abstract

Merging paired-end shotgun reads generated on high-throughput sequencing platforms can substantially improve various subsequent bioinformatics processes, including genome assembly, binning, mapping, annotation, and clustering for taxonomic analysis. With the inexorable growth of sequence data volume and CPU core counts, the speed and scalability of read-processing tools becomes ever-more important. The accuracy of shotgun read merging is crucial as well, as errors introduced by incorrect merging percolate through to reduce the quality of downstream analysis. Thus, we designed a new tool to maximize accuracy and minimize processing time, allowing the use of read merging on larger datasets, and in analyses highly sensitive to errors. We present BBMerge, a new merging tool for paired-end shotgun sequence data. We benchmark BBMerge by comparison with eight other widely used merging tools, assessing speed, accuracy and scalability. Evaluations of both synthetic and real-world datasets demonstrate that BBMerge produces merged shotgun reads with greater accuracy and at higher speed than any existing merging tool examined. BBMerge also provides the ability to merge non-overlapping shotgun read pairs by using *k*-mer frequency information to assemble the unsequenced gap between reads, achieving a significantly higher merge rate while maintaining or increasing accuracy.

## Introduction

Many sequencing platforms–including Illumina and Ion Torrent, which comprise the majority of sequencing capacity at many institutions—produce relatively short reads with tens to low hundreds of bases. Short read lengths result from the decline of signal intensity and integrity with each subsequent base during the sequencing process. To compensate for this, paired-end reads are generated by sequencing two end regions of a nucleic acid fragment [1].

Although many advances have been achieved using paired-end sequencing, there remain situations in which single, longer reads are preferable to paired shorter reads, such as de novo assembly contig-building, read binning or clustering, gene annotation, and small-variant calling. To address this need, several programs have been designed to merge paired short reads into single longer reads; however, most of these are designed to primarily merge 16S rRNA gene amplicon sequences rather than shotgun sequence data.

In this study, we describe BBMerge, a new overlap-based tool for merging short high-throughput shotgun sequencing reads. BBMerge allows simple adjustment of merging sensitivity to accurately and efficiently process large datasets from a variety of sequence types. We designed BBMerge to address common difficulties associated with paired-end shotgun read merging, *i*.*e*. reducing incorrect merge rates, increasing scalability, and handling non-overlapping pairs from longer fragments, which most tools cannot merge. BBMerge’s performance is compared to existing read merging tools that allow shotgun read input using both synthetic and real-world data from *Chlamydomonas reinhardtii* and a defined microbial community with bacterial and archaeal members (MBARC-26) [2], respectively.

## Materials and methods

### Synthetic and real-world sequence data

In order to evaluate merging performance, we used synthetically generated data from a eukaryotic genome to allow precise evaluation of merging accuracy as well as real-world shotgun metagenome data from a prokaryotic community. These two datasets include eukaryotic, bacterial and archaeal organisms with complete reference genomes spanning a large spectrum of %GC.

We synthetically generated 20 million reads based on the *Chlamydomonas reinhardtii* genome (v3.0), which was retrieved from the JGI Plant Genomics Resource Phytozome (ftp://ftp.jgi-psf.org/pub/JGI_data/Chlamy/v3.0/Chlre3.fasta.gz). Synthetic reads were generated using BBMap (https://sourceforge.net/projects/bbmap/) as follows: first, reference sequences were indexed (Table 1A). Second, synthetic reads were generated (Table 1B). Third, read headers were renamed according to their known insert size, to allow subsequent grading (Table 1C). Fourth, reads were decompressed and moved to ramdisk (Table 1D).

**Table 1:** Test data setup.

Step	Note
A	bbmap.sh ref = chlamy_reference.fasta.qz
B	randomreads.sh reads = 20m out = synth20m.fq.gz len = 150 paired int pigz = 32 zl = 6 minq = 14 midq = 24 maxq = 34 qv = 6 adderrors = t nrate = 0.00 maxns = 2 maxnlen = 8 ow mininsert = 100 maxinsert = 400 gaussian overlap = 150 banns fragadapter = GACGCTGCCGACGAATAGAGAGGTGTAGATCTCGGTGGTCGCCGTATCATT fragadapter2 = CCGAGCCCACGAGACTAAGGCGAATCTCGTATGCCGTCTTCTGCTTG
C	rename.sh in = synth20m.fq.gz out = renamed20m.fq.gz renamebyinsert int
D	reformat.sh in = renamed20m.fq.gz out = /dev/shm/r#.fq
E	bbmap.sh ref = mock_ref.fa
F	bbmap.sh in = mock_raw.fq.gz outm = mapped_renamed_noindels.fq.gz ow po indelfilter = 0 renamebyinsert maxindel = 20 minid = 0.75
G	reformat.sh in = clean.fq.gz out = 20m.fq.gz srt = 20m
H	grademerge.sh in = merged.fq
I	reformat.sh in = mock_raw.fq.gz out = r#.fq srt = 20m
J	spades.py—meta -k25,55,95,125—phred-offset 33 -s merged.fq -1 unmerged1.fq -2 unmerged2.fq -o spades_out
K	quast.py -o quast_out -R mock_ref.fa -f spades_*/contigs.fasta

BBMap, BBMerge, RandomReads, Rename, Reformat, and GradeMerge are part of the open-source BBMap package (https://sourceforge.net/projects/bbmap/).

Real-world data is comprised of shotgun metagenomic sequence data from MBARC-26, a microbial mock community consisting of 23 bacterial and 3 archaeal strains [3–10]. DNA extraction from MBARC-26, Illumina metagenome library creation, and shotgun sequencing were performed as described in [4], yielding 2x150 bp reads.

Reference genomes for MBARC-26 were retrieved from JGI’s IMG [11] and used for mapping as described in the following: Reference genomes were first indexed (Table 1E). Second, shotgun metagenome reads were mapped to reference sequences to a) determine insert sizes, and b) to remove reads that mapped with indels or that did not map in a properly paired orientation (Table 1F) using BBMap’s default settings. This filtering step ensured the correct determination of the insert size for each read pair for subsequent grading; insert sizes of unpaired reads cannot be determined, and reads mapped with indels yield a different insert size as calculated by mapping versus merging. Mapping was not necessary for the synthetic data as the true insert size was known *a priori*. The remaining shotgun metagenome reads were subsampled to 20 million read pairs (Table 1G).

Grading was performed using GradeMerge (Table 1H) to obtain the number of correctly and incorrectly merged reads. A merged read was considered correct if its length exactly matched the insert size indicated by its header. The reported percentage values and signal-to-noise ratio (SNR) are defined as:

$$C\%=100*\frac{C}{P}$$ (1)

$$I\%=100*\frac{I}{P}$$ (2)

$$SNR=10\cdot {log}_{10}\frac{C}{I}$$ (3),

where:

a ***C*** is the number of correctly merged reads.
b ***I*** is the number of incorrectly merged reads.
c ***C%*** is the percent of correctly merged reads.
d ***I%*** is the percent of incorrectly merged reads.
e ***P*** is the number of input read pairs.

Assembly quality was evaluated using raw shotgun metagenomic reads from MBARC-26 subsampled to 20 million read pairs (Table 1I). To eliminate potential impact originating from pre-processing, reads were not filtered or trimmed. Reads were merged with each tool, then both the merged and unmerged output was passed to SPAdes v. 3.8.2 [12] for assembly in metagenome mode (Table 1J). Assembled contigs were compared to the metagenome reference using QUAST v. 4.2 [13] for evaluation (Table 1K). Global and local misassemblies as defined in [13] were combined and are reported as “total misassemblies”.

#### Paired-end read merging tools

All algorithms for read merging compared here (Table 2) are based on overlap detection [14–19], with the exception of leeHom [20] and BBMerge, which additionally use adapter-sequence detection; and COPE [18] and BBMerge, which additionally use kmer counts in non-default modes. All tools were executed as described in S1 Table.

**Table 2:** Read merging tools compared in this study in alphabetical order.

Program	Language	Open-Source	Multi-threaded	gzip I/O	Reference
BBMerge v.36.20	Java	Yes	Yes	Yes	^a^
COPE v.1.1.3	C++	Yes	No	Partial	[18]
fastq-join v.1.1.2–537	C++	Yes	No	Yes	[19]
FLASH v.1.2.11	C	Yes	Yes	Yes	[14]
leeHom (retrieved July 15, 2016)	C++	Yes	No	Yes	[20]
PEAR v.0.9.6	C	Yes	Yes	Yes	[17]
Stitch (retrieved July 15, 2016)	Python	Yes	Yes	No	[21]
USEARCH v.8.1.1861	Unknown	No	Yes	No	[15]
XORRO v.0.98	C	Yes	No	No	[16]

^a^: https://sourceforge.net/projects/bbmap/

Although an effort was made to compare all available overlap-based read merging tools for a comprehensive evaluation in this study, the testing methodology precluded the use of PANDAseq [22], which cannot process reads with renamed headers. Eloper [23] was tested, but not included, as it was unable to produce fastq files or retain the original read headers.

#### Parameters and testing

Each program was tested for speed, accuracy, and scalability. All testing was executed on the NERSC Genepool cluster (http://www.nersc.gov/users/computational-systems/genepool/), using a 1 TB, 32-core node based Intel Xeon E5-4650L CPUs @ 2.60GHz. Reads and writes were all performed using a ramdisk to eliminate any impact of contention for the cluster’s shared file system.

Execution of merging tools was performed according to each program’s defaults, except as noted (S1 Table). For accuracy testing, each program was run multiple times; the single parameter that was identified to impact the respective tool’s sensitivity most was varied between runs (if available) (S2 Table). After each run, the resulting output was graded, *i*.*e*. each merged read’s length was compared to the true insert size noted in that read’s header.

Speed and scalability testing was executed using the Linux “time” command, *e*.*g*. “time bbmerge.sh *<other options>*”, with default parameters and varying numbers of threads. For BBMerge, three modes were included in this study: default, REM, and RSEM, as described in 2.2.3 and 2.2.4. For COPE, two modes were included: default (M0), using simple overlap only, and M3, using *k*-mers to join non-overlapping pairs. COPE’s M1 mode was not found to differ substantially from M0, and M2 did not produce output, so neither are included. Speed tests were performed on both synthetic and real-world shotgun metagenome reads. Since no significant difference was found, we only report test results for the real-world metagenome data.

## Results

### BBMerge overlap-detection

Overlap-detection involves multiple heuristics, controlled by constants denoted C_i_. These have already been optimized through extensive empirical testing and do not need to be adjusted by the user; they are only presented to describe the algorithm. For each read pair:

1 Read 2 is reverse-complemented, because read 1 and read 2 are produced from opposite strands of the initial DNA fragment.
2 Read 1 and read 2 are aligned in every possible offset.
  - An “offset” is defined by the relative start position of the reads. For offset ***O*** = 0, each base number ***X***_***i***_ of read 1 aligns to base number ***X***_***i***_ of read 2. In general, each base ***X***_***i***_ in read 1 aligns to base ***X***_***i+O***_ in read 2.
  - This alignment only counts matches and mismatches; indels are not allowed.
3 The standard mode for determining the offset is called “ratio mode”. For each offset, a ratio ***R*** is calculated:
  $$R=\frac{B+C_{0}}{B+G}$$ (4),
  where:

***B*** is the number of mismatches, ***G*** is the number of matches, ***C***_***0***_ is a constant. An optional flag, “ouq”, allows ***B*** and ***G*** to be calculated using quality scores, but this is only helpful if the quality scores are accurate.

**Fig 1:**
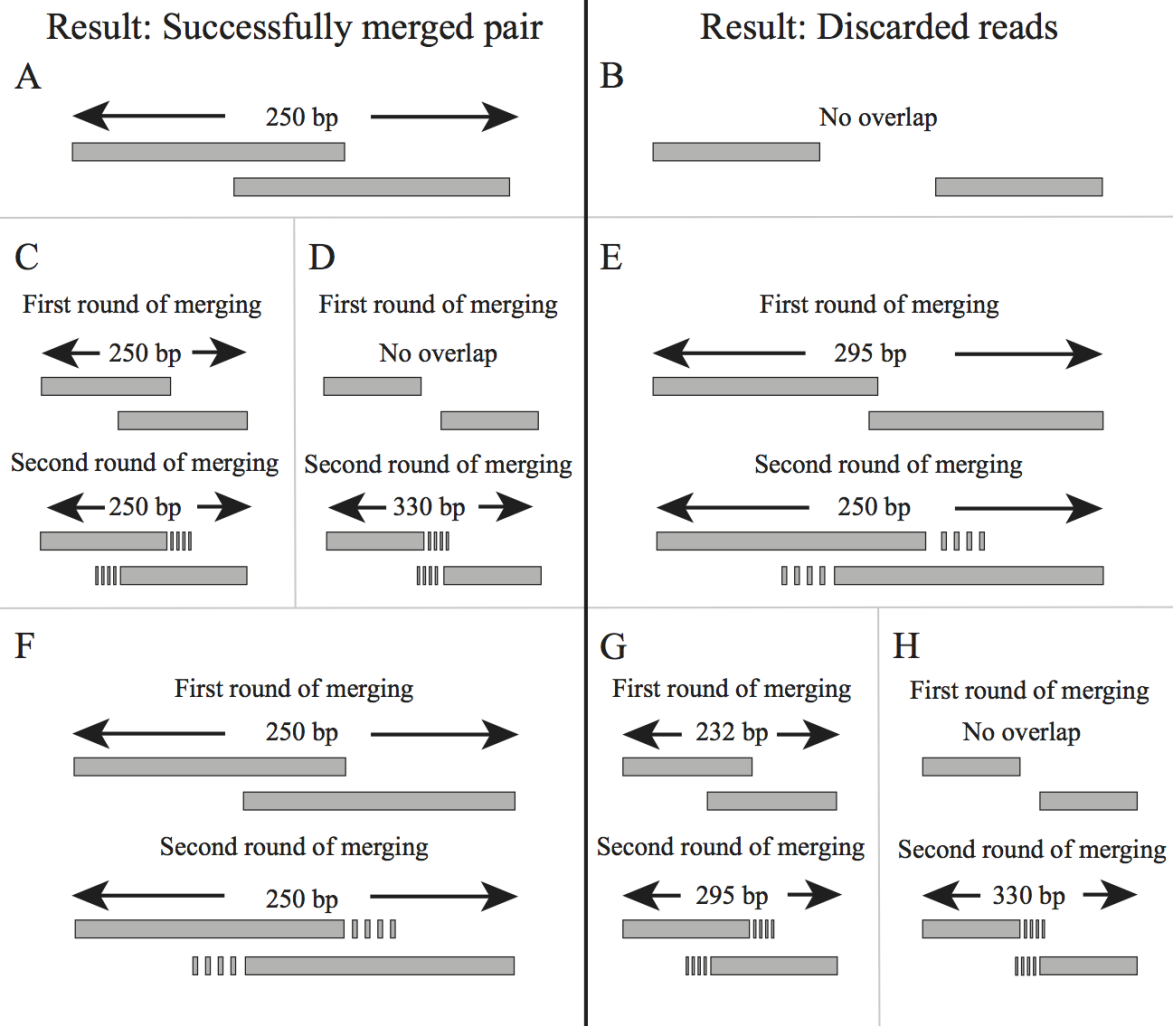
**Merging scenarios in BBMerge modes: default (A-B), REM (C-F), and RSEM (G-I).** The left column (Fig 1A,C,D,F) displays scenarios resulting in successfully merged reads, while the right column (Fig 1B,E,G,H) displays scenarios resulting in discarded unmerged pairs.

4 The two best (lowest) ratios, ***R***_***1***_ and ***R***_***2***_, are tracked throughout the process.
5 Once the alignments finish, ***R***_***1***_ and ***R***_***2***_ are examined to decide whether an alignment will be accepted (Fig 1A) or discarded (Fig 1B), using heuristics with different constants.
  - If ***R***_***1***_**>*C***_***1***_, the alignment will be rejected as invalid.
  - If ***R***_1_*C_***2***_**>*R***_***2***_, the alignment will be rejected as ambiguous.
  - If ***R***_***2***_**<*C***_***3***_, the alignment will be rejected as ambiguous.
  - If ***G*<max(*C***_***4***_, ***V***) the alignment will be rejected as having too short of an overlap. ***V*** is derived from the sequence complexity of a given pair, decreasing as complexity increases.
  - If ***S***<***C***_***6***_, the alignment will be rejected as too short. ***S*** is the insert size implied by the alignment.
  - Otherwise, the best alignment will be reported for further consideration.
6 At extreme sensitivity settings, an additional algorithm–“flat mode”–is used. This mode determines the best overlap by minimizing the number of mismatching bases.
  - At the “xstrict” and “ustrict” settings, the alignment is only accepted if the best offset from flat mode matches the best offset from ratio mode.
  - At the “xloose” setting, an alignment produced by flat mode will be accepted if no alignment was produced by ratio mode.
  - Otherwise, flat mode is not used.
7 If the pair has an alignment reported in 5) or 6), it is subjected to further scrutiny.
  - If the implied insert size is shorter than the read length, and adapter sequences have been specified, non-overlapping portions of the reads are aligned to respective expected adapter sequence. If they do not match, the alignment is rejected.
  - The number of expected mismatches (***E***) in the overlap is calculated using quality scores. If *B*>E*C_***5***_, the alignment is rejected.
  - The probability (***P***) of the specific pattern of matches and mismatches is calculated. If ***P***<***C***_***6***_, the alignment is rejected.
8 If, at this point, the alignment has not been rejected, the read pair is merged to create a new read of size equal to the insert size implied by the overlap.
  - The overlapping portions of the reads are represented in the resulting read as a consensus of the two parent sequences. Matching bases are assigned an increased quality score; for non-matching bases, the base with the higher quality score is used, and is assigned a quality score equal to the difference between the two parent qualities. Where both quality scores are equal and the bases mismatch, the resulting base is N.
  - If only the tail ends of the reads overlap, the insert size (and thus resulting read) is longer than the original read length. The merged read will be composed of the non-overlapping portion of read 1; the consensus of the overlapping sequence; and the non-overlapping portion of read 2, respectively.
  - If the tail ends of the reads do not overlap, the insert size is shorter than the initial read length, and the non-overlapping portion is non-genomic sequencing adapter read-through. In this case the resulting read is trimmed to the insert size, and will be 100% consensus sequence.

### BBMerge *k*-mer-based modes

**Fig 2:**
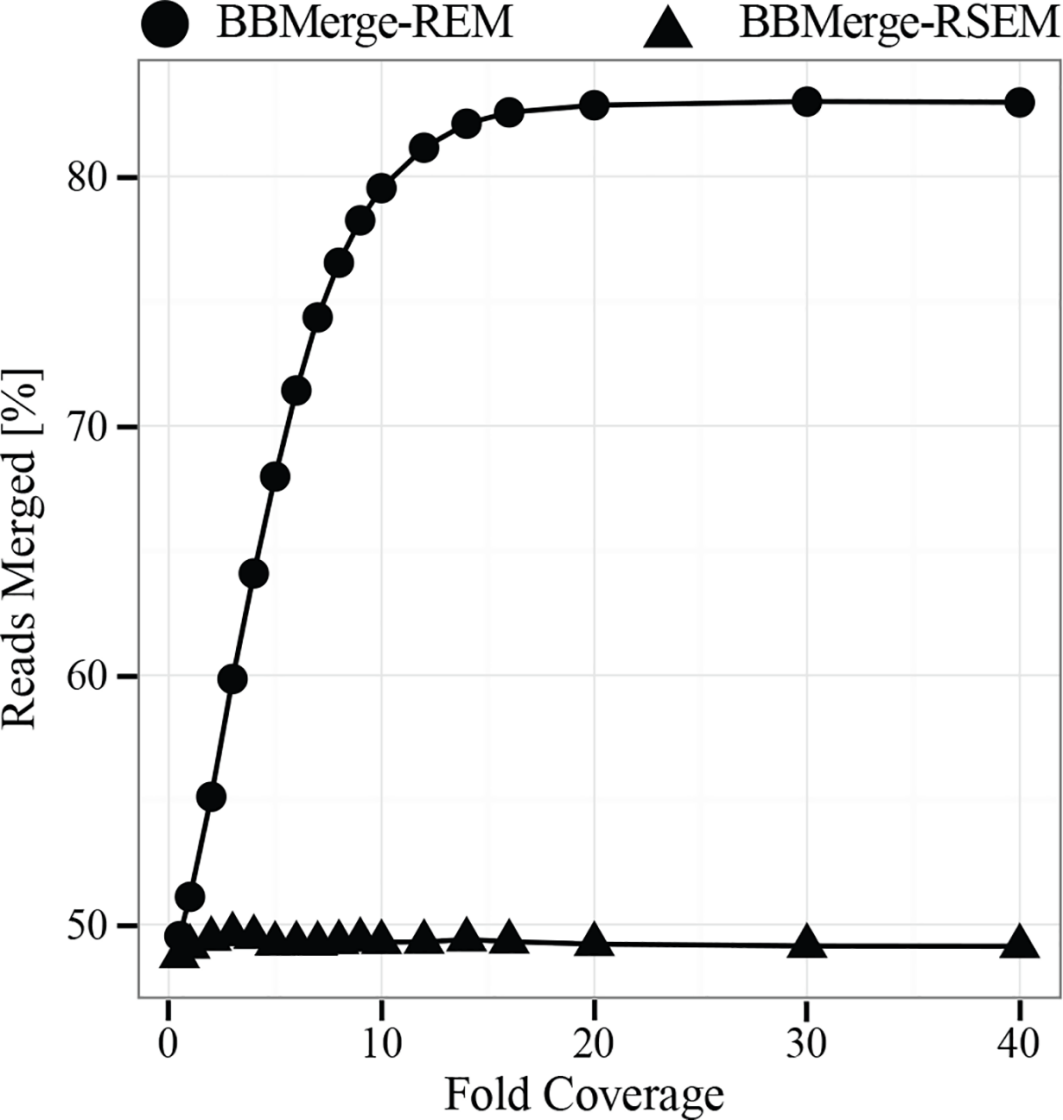
Relationship between % merged reads and genome coverage.

BBMerge has the ability to improve merging accuracy or merge non-overlapping reads using *k*-mer frequency information, if the sequencing depth is sufficient (Fig 2) and the library is randomly sheared. There are two *k*-mer-using modes described in this paper, REM and RSEM, which stand for “Require Extension Match” and “Require Strict Extension Match". In each case, the default BBMerge algorithm is used with an additional *k*-mer-based extension step. To summarize: The input read file is processed once, to build a table of *k*-mer counts. The file is then processed a second time to perform merging. Steps performed during the merging phase for each read pair include:

1. The standard BBMerge algorithm is used to determine the insert size ***S***_***0***_ based purely on overlap (Fig 1A and 1B).
2. Each read is extended by a fixed length on the tail end only, using the Tadpole assembler (https://sourceforge.net/projects/bbmap/). When not specified, as in this study, extension defaults to 50 bp. Extension will stop prematurely if a branch *k*-mer is encountered, or *k*-mer depth drops below a set threshold, so extension may not reach the full length specified by the user.
  - A “branch *k*-mer” is a *k*-mer with more than one possible next *k*-mer. They are identified based on BBMerge’s optional Tadpole-specific parameters.
  - If extension completely fails such that neither read is extended by at least one base, insert size ***S***_***0***_ is used regardless of mode and subsequent steps are skipped.
3. If extension was successful, the BBMerge algorithm is applied to the extended reads to obtain a new insert size ***S***_***1***_.
4. In REM mode, the alignment is accepted if ***S***_***0***_ **= *S***_***1***_ (Fig 1C). If there is no ***S***_***0***_ because overlap failed in step 1, ***S***_***1***_ will be used (Fig 1D). If ***S***_***0***_ and ***S***_***1***_ exist and ***S***_***0***_**! = *S***_***1***_, the alignment is rejected (Fig 1E).
5. In RSEM mode, the alignment is exclusively accepted if ***S***_***0***_ **= *S***_***1***_ (Fig 1F). If ***S***_***0***_***≠S***_***1***_ (Fig 1G), or if there was no initial overlap detected (Fig 1H), the alignment is rejected.

In practice, REM mode can produce merged reads from initially non-overlapping pairs, with insert size > sum of the read lengths. RSEM will only produce merged reads < sum of the read lengths–a strict subset of the merged reads produced by BBMerge run in pure overlap mode. Requiring that the overlap after extension matches the initial overlap reduces false-positive merges caused by short repeats.

Although *k*-mer-based modes can increase accuracy and merge rates, read processing requires more time and memory in these modes. This memory constraint may hence render *k*-mer modes impractical on very large datasets. Though not evaluated in this study, BBMerge also has additional *k*-mer-related options, “ecct” and “kfilter”. “ecct” enables *k*-mer-based error correction of reads that initially fail to merge; if the reads still fail to merge after correction, the changes are rolled back. This can increase the merge rate in data with many sequencing errors. “kfilter” is a setting applied after a potential overlap is found; if the merged read contains any *k*-mers that were not already present at a specified depth in the original file, the overlap is assumed to be wrong and will be rejected. All *k*-mer-using modes use the same *k*-mer count table, so they can be enabled concurrently without using additional memory, and with little speed impact.

### BBMerge threading

BBMerge uses both pipelined and parallel threads to achieve a high degree of scalability. Data is streamed from and to disk during execution, so that BBMerge’s memory requirements (in default overlap mode) are unrelated to the amount of input data. Data is read by one thread per file and packaged into lists of ***P*** read pairs each (***P*** = 200 by default). These lists are added to an ArrayBlockingQueue, a data structure that allows safe concurrent read/write access. A number of parallel worker threads is spawned (controlled by the “t” flag). Each worker fetches a list of reads from the queue; if the queue is empty, it will block until a new list is added. The worker thread will then iterate through the list and attempt to merge each of the read pairs, tracking statistics in thread-local variables, and adding merged reads to a new list. The finished list of merged reads is added to an output ArrayBlockingQueue, which is being fed by all of the worker threads. An output thread pulls lists from this output queue, and writes the reads to disk. The worker threads finish when all reads have been processed. Finally, the master thread summarizes and prints the statistics from the worker threads. As a result, the worker threads do not interfere with memory used by any other thread except when pulling lists from the input queue, or sending lists to the output queue; this means shared memory is only mutated twice per ***P*** read pairs. Furthermore, ***P*** can be set to an arbitrarily high value on the command line (with the “readbufferlength” flag), so that distributing and gathering work has minimal negative impact on scalability. Most tools in the BBMap package share this threading design.

### Deployment and use

BBMerge is written in Java, with no other dependencies. It is distributed with both the source and precompiled class files, allowing simple deployment and use on any computer supporting Java, from Windows laptops to HPC Linux-based clusters. BBMerge is designed for production use, so to simplify pipeline integration, it supports a wide variety of input and output formats–fasta or fastq; interleaved or dual-file; raw or compressed; encoded in ASCII-33 or ASCII-64, with input format autodetection. It also provides alternative processing modes such as insert-size histogram generation, adapter-sequence detection, and overlap-based error-correction (without merging), allowing its use in situations when paired reads are preferred over merged reads.

## Discussion

We tested BBMerge in three modes (default, REM, RSEM) and compared its merging performance with eight other read merging programs (Table 2) using synthetically generated reads from an algae genome, and real-world shotgun metagenomic reads from a prokaryotic mock community (MBARC-26). Merging performance was evaluated based on accuracy, speed and computing efficiency.

### Accuracy of paired-end read merging

**Fig 3:**
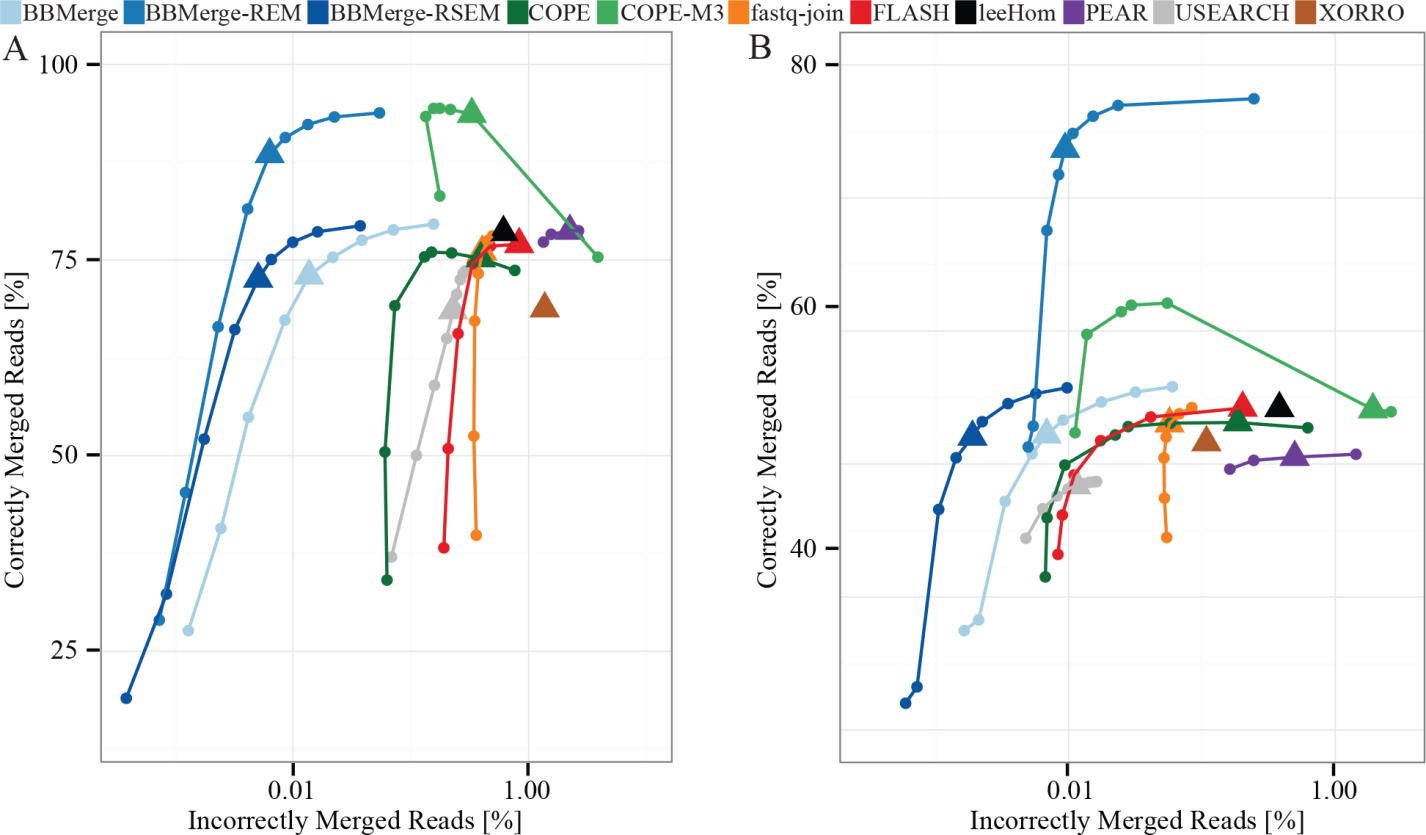
**Comparison of merging accuracy by program using synthetic (A) and shotgun metagenome sequences (B).** Correctly merged reads are defined as % of total input pairs. Program performance at default sensitivity is indicated by a triangle.

BBMerge outperformed all other tools in merging accuracy across the sensitivity curve, with the lowest rate of incorrectly merged reads for any given rate of correctly merged reads, though this difference was more pronounced in the synthetic (Fig 3A) compared to the real-world data (Fig 3B). Similarly, BBMerge resulted in the highest correct merge rate (Fig 3) of all non-*k*-mer-using tools.

Results from the three discussed *k*-mer-utilizing modes are clearly distinguishable from those of the purely overlap-based tools and modes (Fig 3). BBMerge’s RSEM mode substantially reduced the rate of incorrectly merged reads, while slightly reducing the rate of correctly merged reads. BBMerge’s REM mode, and COPE’s M3 mode, substantially increased correct merge rates compared to the programs’ default modes by merging initially non-overlapping reads (Fig 3). BBMerge-REM achieved the highest rate of correctly merged reads in the real-world data (77.5%) followed by COPE-M3 (62.1%), and COPE-M3 achieved the highest merge rate in the synthetic data (94.4%) followed by BBMerge-REM (93.8%). Stitch yielded 69.2% incorrectly and 0.8% correctly merged reads in the synthetic data, and 49.1% incorrectly and 0.64% correctly merged reads in the real-world data (S3 Table).

### Speed and scalability of paired-end read merging

**Fig 4:**
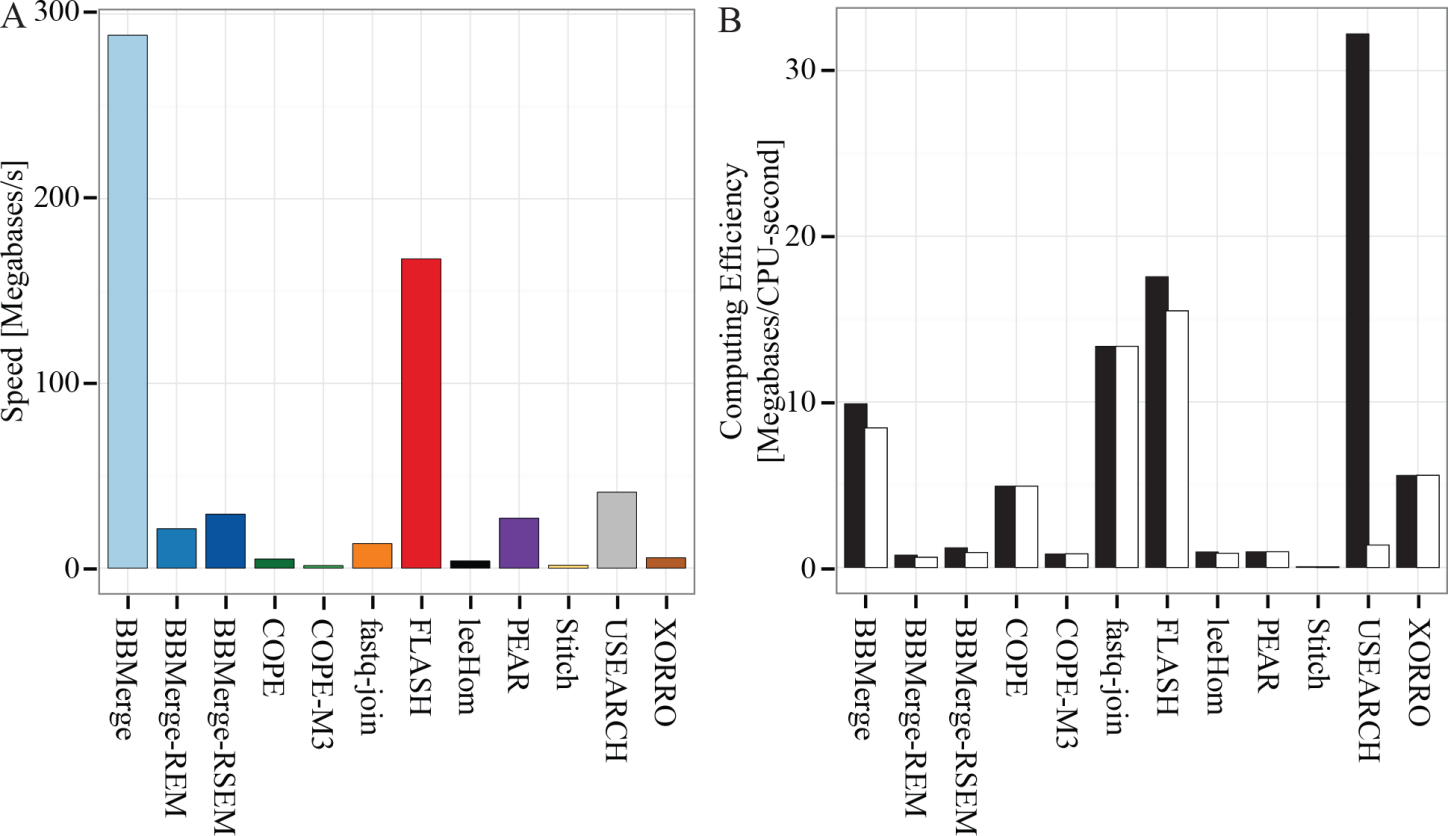
Speed comparison by program of shotgun metagenome sequences.

Merging speeds were evaluated using the real-world metagenome reads and programs set to default sensitivity. Multi-threaded programs were allowed to use all 32 available threads. Compared to the other merging tools, BBMerge and FLASH were substantially faster, although we found that USEARCH, PEAR, BBMerge REM/RSEM, and fastq-join can all merge large datasets within reasonable timescales (Fig 4). Based on the performance on our shotgun sequence datasets, XORRO, COPE, leeHom and Stitch were projected to require >1 day to process a 500 Gbp dataset.

**Fig 5:**
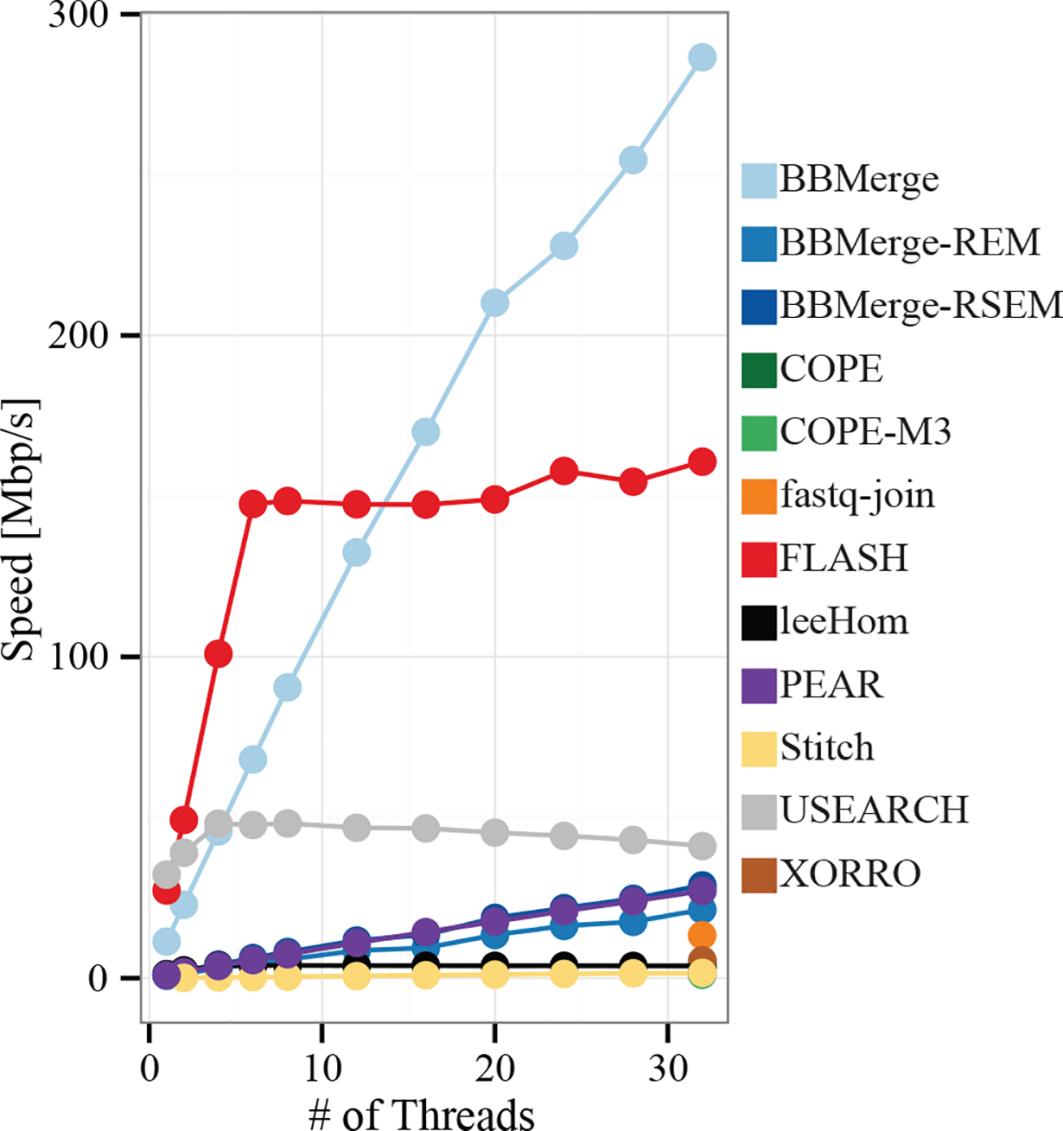
Scalability of each program, determined by measuring speed using various numbers of threads.

BBMerge variants, PEAR, and Stitch exhibited near-perfect scaling in these tests, and are expected to continue scaling past 32 threads if run on a system with more CPU cores (Fig 5). FLASH scaled linearly to 6 threads, at which point speed plateaued. leeHom scaled to a peak at 4 threads, after which speed slightly declined. USEARCH also reached a peak at ~4 threads, but did not scale as well; 4-threaded speed was only 150% of single-threaded speed, rather than an ideal 400%. Subsequently, USEARCH’s performance declined, ending at 85% of its peak speed at the maximum of 32 threads. Single-threaded programs (fastq-join, XORRO, and COPE) are each represented by a single point.

### Assembly quality following read merging

**Fig 6:**
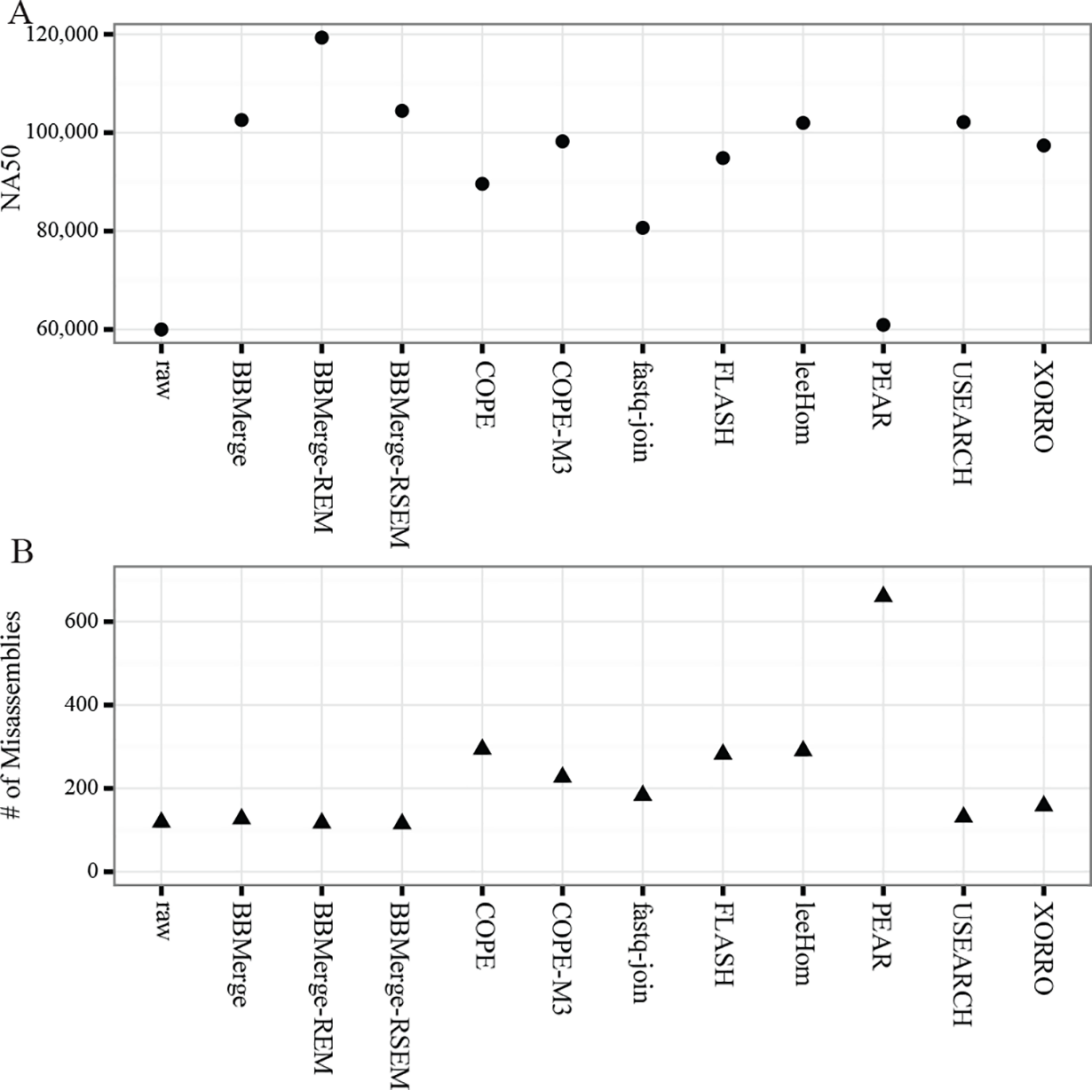
NA50 length and misassembly rates for a SPAdes assembly of each program’s output at default settings.

Assembly quality was evaluated with QUAST; we report here assembly continuity (NA50), genome completeness, misassemblies, and indels as defined in [13] (Table 3, S4 Table). Gurevich *et al*. [13] defined NA50 as the length at which the collection of all reference-aligned contigs, of that length or longer, contain at least half of the assembled bases. Merged reads were generally characterized by substantially improved assembly continuity compared to the raw data (Table 3, Fig 6A), with BBMerge-REM reaching a nearly two-fold increase in NA50 (119 kbp compared to 60 kbp). BBMerge-RSEM, BBMerge, USEARCH, and leeHom resulted in similar NA50 metrics (101–104 kbp). The NA50 achieved with the remaining programs ranged from 61 kbp (PEAR) to 98 kbp (COPE-M3), aside from Stitch at 5.6 kbp. The raw data resulted in a total misassembly count of 119. Only BBMerge-RSEM and BBMerge-REM reduced this count, to 115 and 117, respectively (Table 3, Fig 6B). The studied merge tools fell into 3 misassembly-count clusters: BBMerge variants and USEARCH ranged from 115 to 131; XORRO, fastq-join, COPE-M3, FLASH, leeHom, and COPE ranged from 158 to 294; and PEAR and Stitch resulted in 660 and 20,986 misassemblies, respectively.

**Table 3:** Assembly metrics reported by QUAST for SPAdes metagenomic assemblies.

Program	NA50 (bp)	Total Misassemblies	Indels/ 100 kbp	Genome Completeness (%)
Raw Data	60007	119	1.13	84.5
BBMerge	102577	127	0.84	84.88
BBMerge-REM	119328	117	0.81	85.18
BBMerge-RSEM	104441	115	0.84	84.88
COPE	89603	294	1.52	85.17
COPE-M3	98240	227	1.24	83.92
fastq-join	80672	183	1.17	84.74
FLASH	94846	282	1.41	85.20
leeHom	101992	290	1.1	84.91
PEAR	60937	660	1.46	84.28
Stitch	5623	20986	47.78	68.38
USEARCH	102156	131	0.88	84.77
XORRO	97403	158	1.08	84.85

Indel rates are noted because they can induce frameshifts, which disrupt gene annotation. BBMerge variants and USEARCH clustered together closely, with rates ranging from 0.81 (BBMerge-REM) to 0.88 (USEARCH) indels per 100 kbp (Table 3). The other tools yielded rates ranging from 1.08 (XORRO) to 1.52 (COPE), except for Stitch (47.78 per 100 kbp). The raw data yielded 1.13 indels per 100 kbp. The fraction of reference bases covered by assemblies exhibited a narrow range from 83.9% (COPE-M3) to 85.2% (FLASH), aside from Stitch at 68.4% (Table 3). All tools except PEAR, COPE-M3, and Stitch exceeded the 84.5% genome coverage of the raw read assembly. BBMerge-REM outperformed BBMerge in every assembly metric, but COPE-M3’s performance relative to COPE was more nuanced: COPE-M3 had a greater NA50 and fewer misassemblies and indels, but a 1.2% lower genome recovery than COPE.

## Conclusion

Correctly merged shotgun reads can improve the performance of applications that benefit from longer reads, yet erroneously merged reads can create serious issues due to the introduction of new errors, a concern that is not present for other common preprocessing steps such as quality-trimming. Even at a low rate, the addition of incorrectly merged reads can cause misassemblies and reduced assembly contiguity compared to unmerged or correctly merged data (Fig 6). It is this possibility of introducing new errors that renders merging especially sensitive to accuracy.

Since BBMerge has been developed primarily as a tool to aid in clustering and de-novo assembly of shotgun metagenome sequence data, minimizing the false-positive merge rate has been considered paramount. Our data indicates that BBMerge successfully minimized the false-positive rate when merging shotgun reads from synthetic and real-world datasets, and was able to improve assembly quality by increasing continuity while reducing the number of misassemblies. Its ability to achieve maximal accuracy while scaling near-linearly to reach the highest speed of the compared software makes BBMerge a promising tool for improving the assembly of large datasets such as shotgun metagenomes.

## Supporting information

S1 Table Program command lines. Non-default parameters are stated in bold letters.
(DOC)

S2 Table Program sensitivity parameters. Default settings are stated in bold letters.
(DOCX)

S3 Table Number of correctly and incorrectly merged read pairs, and Signal-Noise Ratio (SNR), from the synthetic **(A)** and real-world **(B)** shotgun datasets by program and sensitivity. All numbers are out of 20,000,000 input read pairs. Defaults are in bold.
(DOCX)

S4 Table Assembly report by program. (DOC)
