# Supplementary material for: BBMerge – Accurate paired shotgun read merging via overlap
Source: PLoS One. 2017 Oct 26;12(10):e0185056. doi: 10.1371/journal.pone.0185056 (PMC5657622; doi:10.1371/journal.pone.0185056)
Supplement: S1 Table — Non-default parameters are stated in bold letters. (DOC) [file pone.0185056.s001.doc]

**S1 Table. Program Command Lines.** Non-default parameters are stated in bold letters.

| Program | Command |
| --- | --- |
| BBMerge | bbmerge.sh in=r#.fq out=merged.fq **adapters=adapters.fa** **t=32** |
| BBMerge-REM | bbmerge-auto.sh in=r#.fq out=merged.fq **adapters=adapters.fa** **t=32** rem **k=62** |
| BBMerge-RSEM | bbmerge-auto.sh in=r#.fq out=merged.fq **adapters=adapters.fa** **t=32** rsem **k=62** |
| COPE | cope -a r1.fq -b r2.fq -o out.fq -2 u1.fq -3 u2.fq -m 0 -s 33 **-u 200** -c 0.75 |
| COPE-M3 | cope -a r1.fq -b r2.fq -o out.fq -2 u1.fq -3 u2.fq -m 3 -s 33 **-u 200** -c 0.75 -t output.freq.cz -f output.freq.cz.len |
| fastq-join | fastq-join -p 8 r1.fq r2.fq -o fqj%.fq |
| FLASH | flash **-t 32** -x 0.25 **-M 200** r1.fq r2.fq |
| leeHom | leeHomMT **-t 32** **--ancientdna** -fq1 r1.fq -fq2 r2.fq -fqo outlh **-f *adapter1* -s *adapter2*** |
| PEAR | pear -p 0.01 **-j 32** -f r1.fq -r r2.fq -o outpear |
| Stitch | stitch.py -i r1.fq -j r2.fq -o out **-t 32** |
| USEARCH | usearch -fastq_mergepairs r1.fq -reverse r2.fq -fastqout out.fq **-threads 32** -fastq_maxdiffpct 5 |
| XORRO | perl xorro_wrapper.pl -i1 r1.fq -i2 r2.fq -f T -o out.fq |
