# Supplementary material for: BBMerge – Accurate paired shotgun read merging via overlap
Source: PLoS One. 2017 Oct 26;12(10):e0185056. doi: 10.1371/journal.pone.0185056 (PMC5657622; doi:10.1371/journal.pone.0185056)
Supplement: S2 Table — Default settings are stated in bold letters. (DOCX) [file pone.0185056.s002.docx]

**S2 Table.** Program Sensitivity Parameters. Default settings are stated in bold letters.

| Program | Parameter Settings |
| --- | --- |
| BBMerge | xloose/uloose/vloose/loose/**default**/strict/vstrict/ustrict/xstrict |
| BBMerge-REM | xloose/uloose/vloose/loose/**default**/strict/vstrict/ustrict/xstrict |
| BBMerge-RSEM | xloose/uloose/vloose/loose/**default**/strict/vstrict/ustrict/xstrict |
| COPE | -c 0.7/**0.75**/0.8/0.85/0.9/0.95/0.98/1 |
| COPE-M3 | -c 0.7/**0.75**/0.8/0.85/0.9/0.95/0.98/1 |
| fastq-join | -p 1/2/4/6/**8**/10/12 |
| FLASH | -x 0.01/0.02/0.04/0.08/0.16/**0.25** |
| leeHom | *None* |
| PEAR | -p 0.0001/0.001/**0.01**/0.05/1 |
| Stitch | *None* |
| USEARCH | -fastq_maxdiffpct 1/2/3/4/**5**/6/8/10/12 |
| XORRO | *None* |
