# Supplementary material for: BBMerge – Accurate paired shotgun read merging via overlap
Source: PLoS One. 2017 Oct 26;12(10):e0185056. doi: 10.1371/journal.pone.0185056 (PMC5657622; doi:10.1371/journal.pone.0185056)
Supplement: S3 Table — Number of correctly and incorrectly merged read pairs, and Signal-Noise Ratio (SNR), from the synthetic (A) and real-world (B) shotgun datasets by program and sensitivity. All numbers are out of 20,000,000 input read pairs. Defaults are in bold. (DOCX) [file pone.0185056.s003.docx]

**S3 Table.**Number of correctly and incorrectly merged read pairs, and Signal-Noise Ratio (SNR), from the synthetic **(A)** and real-world **(B)** shotgun datasets by program and sensitivity. All numbers are out of 20,000,000 input read pairs. Defaults are in bold.

A

| Program | Correct | Incorrect | SNR | Sensitivity Parameter |
| --- | --- | --- | --- | --- |
| BBMerge | 15919074 | 31135 | 27.095 | xloose |
| BBMerge | 15772156 | 14197 | 30.461 | uloose |
| BBMerge | 15509467 | 7677 | 33.056 | vloose |
| BBMerge | 15072021 | 4354 | 35.394 | loose |
| **BBMerge** | **14599654** | **2738** | **37.27** |  |
| BBMerge | 13463211 | 1709 | 38.965 | strict |
| BBMerge | 10983358 | 838 | 41.175 | vstrict |
| BBMerge | 8142191 | 491 | 42.197 | ustrict |
| BBMerge | 5527665 | 259 | 43.293 | xstrict |
| BBMerge-rem | 18761155 | 10838 | 32.386 | xloose |
| BBMerge-rem | 18658124 | 4484 | 36.193 | uloose |
| BBMerge-rem | 18464788 | 2678 | 38.386 | vloose |
| BBMerge-rem | 18132127 | 1722 | 40.225 | loose |
| **BBMerge-rem** | **17703409** | **1267** | **41.453** |  |
| BBMerge-rem | 16305510 | 825 | 42.959 | strict |
| BBMerge-rem | 13294611 | 463 | 44.581 | vstrict |
| BBMerge-rem | 9057803 | 245 | 45.679 | ustrict |
| BBMerge-rem | 5798165 | 147 | 45.96 | xstrict |
| BBMerge-rsem | 15875570 | 7439 | 33.294 | xloose |
| BBMerge-rsem | 15722734 | 3230 | 36.874 | uloose |
| BBMerge-rsem | 15455880 | 1989 | 38.905 | vloose |
| BBMerge-rsem | 15014061 | 1310 | 40.593 | loose |
| **BBMerge-rsem** | **14518766** | **1014** | **41.559** |  |
| BBMerge-rsem | 13222702 | 642 | 43.138 | strict |
| BBMerge-rsem | 10422884 | 353 | 44.702 | vstrict |
| BBMerge-rsem | 6463935 | 169 | 45.826 | ustrict |
| BBMerge-rsem | 3798377 | 77 | 46.931 | xstrict |
| COPE | 14735047 | 152408 | 19.898 | -c 0.70 |
| **COPE** | **15039917** | **78432** | **22.85** | **-c 0.75** |
| COPE | 15181052 | 44360 | 25.356 | -c 0.80 |
| COPE | 15202565 | 29981 | 27.059 | -c 0.85 |
| COPE | 15081825 | 26144 | 27.618 | -c 0.90 |
| COPE | 13832484 | 14620 | 29.764 | -c 0.95 |
| COPE | 10095874 | 12058 | 29.234 | -c 0.98 |
| COPE | 6820239 | 12550 | 27.36 | -c 1 |
| Cope-M3 | 15075149 | 771453 | 13.126 | -c 0.70 |
| **Cope-M3** | **18730007** | **65759** | **24.561** | **-c 0.75** |
| Cope-M3 | 18849478 | 43317 | 26.396 | -c 0.80 |
| Cope-M3 | 18878002 | 35160 | 27.307 | -c 0.85 |
| Cope-M3 | 18874763 | 31146 | 27.832 | -c 0.90 |
| Cope-M3 | 18669556 | 26742 | 28.446 | -c 0.95 |
| Cope-M3 | 16637065 | 35193 | 26.755 | -c 1 |
| fastq-join | 7966277 | 71738 | 20.494 | -p 1 |
| fastq-join | 10502075 | 68401 | 21.89 | -p 2 |
| fastq-join | 13441550 | 69572 | 22.883 | -p 4 |
| fastq-join | 14653168 | 74591 | 22.955 | -p 6 |
| **fastq-join** | **15210436** | **80687** | **22.776** | **-p 8** |
| fastq-join | 15496406 | 90307 | 22.37 | -p 10 |
| fastq-join | 15615981 | 98464 | 22.03 | -p 12 |
| FLASH | 7644643 | 38065 | 23.05 | -x 0.01 |
| FLASH | 10179163 | 41458 | 23.919 | -x 0.02 |
| FLASH | 13118207 | 50302 | 24.18 | -x 0.04 |
| FLASH | 14889076 | 66315 | 23.532 | -x 0.08 |
| FLASH | 15356319 | 94428 | 22.138 | -x 0.16 |
| **FLASH** | **15403277** | **165950** | **19.723** | **-x 0.25** |
| **leeHom** | **15710859** | **122039** | **21.131** | ***None*** |
| PEAR | 15459266 | 266575 | 17.708 | -p 0.0001 |
| PEAR | 15659596 | 310197 | 17.117 | -p 0.001 |
| PEAR | 15743782 | 446799 | 15.592 | -p 0.01 |
| PEAR | 15749493 | 529191 | 14.88 | -p 0.05 |
| **Stitch** | **166131** | **13836118** | **0.052** | ***None*** |
| USEARCH | 7409859 | 13685 | 27.344 | -fastq_maxdiffpct 1 |
| USEARCH | 10007299 | 22309 | 26.528 | -fastq_maxdiffpct 2 |
| USEARCH | 11797094 | 31694 | 25.72 | -fastq_maxdiffpct 3 |
| USEARCH | 12996098 | 40324 | 25.096 | -fastq_maxdiffpct 4 |
| **USEARCH** | **13712088** | **45656** | **24.79** | **-fastq_maxdiffpct 5** |
| USEARCH | 14121183 | 48847 | 24.625 | -fastq_maxdiffpct 6 |
| USEARCH | 14500366 | 52547 | 24.424 | -fastq_maxdiffpct 8 |
| USEARCH | 14659228 | 55543 | 24.231 | -fastq_maxdiffpct 10 |
| USEARCH | 14721121 | 57896 | 24.07 | -fastq_maxdiffpct 12 |
| **XORRO** | **13759342** | **273250** | **17.106** | ***None*** |

B

| Program | Correct | Incorrect | SNR | Sensitivity Parameter |
| --- | --- | --- | --- | --- |
| BBMerge | 11162052 | 11976 | 29.699 | xloose |
| BBMerge | 11080059 | 6338 | 32.428 | uloose |
| BBMerge | 10930591 | 3515 | 34.929 | vloose |
| BBMerge | 10659957 | 1813 | 37.694 | loose |
| **BBMerge** | **10440019** | **1366** | **38.833** |  |
| BBMerge | 10153142 | 1059 | 39.817 | strict |
| BBMerge | 9438259 | 666 | 41.514 | vstrict |
| BBMerge | 7655296 | 421 | 42.597 | ustrict |
| BBMerge | 7494250 | 328 | 43.589 | xstrict |
| BBMerge-rem | 15491612 | 49118 | 25.002 | xloose |
| BBMerge-rem | 15391500 | 4686 | 35.166 | uloose |
| BBMerge-rem | 15229431 | 3041 | 36.998 | vloose |
| BBMerge-rem | 14970292 | 2148 | 38.433 | loose |
| **BBMerge-rem** | **14735621** | **1875** | **38.954** |  |
| BBMerge-rem | 14349836 | 1676 | 39.326 | strict |
| BBMerge-rem | 13510817 | 1369 | 39.943 | vstrict |
| BBMerge-rem | 10569659 | 1082 | 39.899 | ustrict |
| BBMerge-rem | 10255727 | 991 | 40.149 | xstrict |
| BBMerge-rsem | 11145579 | 1941 | 37.592 | xloose |
| BBMerge-rsem | 11058323 | 1130 | 39.907 | uloose |
| BBMerge-rsem | 10907772 | 699 | 41.933 | vloose |
| BBMerge-rsem | 10636078 | 448 | 43.755 | loose |
| **BBMerge-rsem** | **10406565** | **378** | **44.398** |  |
| BBMerge-rsem | 10093854 | 285 | 45.492 | strict |
| BBMerge-rsem | 9316754 | 211 | 46.45 | vstrict |
| BBMerge-rsem | 6648569 | 145 | 46.614 | ustrict |
| BBMerge-rsem | 6402193 | 119 | 47.308 | xstrict |
| COPE | 10543761 | 124460 | 19.331 | -c 0.70 |
| **COPE** | **10623495** | **37147** | **24.579** | **-c 0.75** |
| COPE | 10613873 | 11583 | 29.625 | -c 0.80 |
| COPE | 10561249 | 5584 | 32.77 | -c 0.85 |
| COPE | 10435048 | 4467 | 33.687 | -c 0.90 |
| COPE | 9983186 | 1868 | 37.28 | -c 0.95 |
| COPE | 9189956 | 1374 | 38.254 | -c 0.98 |
| COPE | 8301458 | 1333 | 37.944 | -c 1 |
| Cope-M3 | 10785064 | 526633 | 13.32 | -c 0.70 |
| **Cope-M3** | **10818835** | **384161** | **14.648** | **-c 0.75** |
| Cope-M3 | 12417859 | 10970 | 30.542 | -c 0.80 |
| Cope-M3 | 12387802 | 5893 | 33.229 | -c 0.85 |
| Cope-M3 | 12289696 | 4956 | 33.946 | -c 0.90 |
| Cope-M3 | 11948629 | 2734 | 36.406 | -c 0.95 |
| Cope-M3 | 10470276 | 2227 | 36.723 | -c 1 |
| fastq-join | 8894650 | 10860 | 29.138 | -p 1 |
| fastq-join | 9487139 | 10449 | 29.585 | -p 2 |
| fastq-join | 10091294 | 10362 | 29.889 | -p 4 |
| fastq-join | 10404764 | 10764 | 29.857 | -p 6 |
| **fastq-join** | **10607687** | **11403** | **29.691** | **-p 8** |
| fastq-join | 10754105 | 13488 | 29.022 | -p 10 |
| fastq-join | 10847571 | 16770 | 28.115 | -p 12 |
| FLASH | 8640512 | 1658 | 37.17 | -x 0.01 |
| FLASH | 9231841 | 1788 | 37.13 | -x 0.02 |
| FLASH | 9835058 | 2195 | 36.514 | -x 0.04 |
| FLASH | 10350982 | 3468 | 34.75 | -x 0.08 |
| FLASH | 10705633 | 8258 | 31.131 | -x 0.16 |
| **FLASH** | **10842946** | **40398** | **24.304** | **-x 0.25** |
| **leeHom** | **10836942** | **76229** | **21.558** | ***None*** |
| PEAR | 9922131 | 32376 | 24.878 | -p 0.0001 |
| PEAR | 10054331 | 48956 | 23.147 | -p 0.001 |
| PEAR | 10102437 | 99401 | 20.113 | -p 0.01 |
| PEAR | 10146133 | 286688 | 15.61 | -p 0.05 |
| **Stitch** | **128361** | **9816120** | **0.056** | ***None*** |
| USEARCH | 8883045 | 953 | 39.695 | -fastq_maxdiffpct 1 |
| USEARCH | 9326480 | 1275 | 38.643 | -fastq_maxdiffpct 2 |
| USEARCH | 9517810 | 1628 | 37.67 | -fastq_maxdiffpct 3 |
| USEARCH | 9622306 | 1975 | 36.878 | -fastq_maxdiffpct 4 |
| **USEARCH** | **9677490** | **2274** | **36.291** | **-fastq_maxdiffpct 5** |
| USEARCH | 9706259 | 2538 | 35.827 | -fastq_maxdiffpct 6 |
| USEARCH | 9725607 | 2866 | 35.308 | -fastq_maxdiffpct 8 |
| USEARCH | 9732517 | 3094 | 34.978 | -fastq_maxdiffpct 10 |
| USEARCH | 9734942 | 3236 | 34.785 | -fastq_maxdiffpct 12 |
| **XORRO** | **10323276** | **21583** | **26.806** | ***None*** |
