# Supplementary material for: BBMerge – Accurate paired shotgun read merging via overlap
Source: PLoS One. 2017 Oct 26;12(10):e0185056. doi: 10.1371/journal.pone.0185056 (PMC5657622; doi:10.1371/journal.pone.0185056)
Supplement: S4 Table — (DOC) [file pone.0185056.s004.doc]

**S4 Table**. Assembly report by program.

| Assembly | raw | BBMerge | BBMerge-REM | BBMerge-RSEM | COPE | COPE-M3 | fastq-join | FLASH | leeHom | PEAR | Stitch | USEARCH | XORRO | |
| --- | --- | --- | --- | --- | --- | --- | --- | --- | --- | --- | --- | --- | --- | --- |
| # contigs (>= 0 bp) | 19607 | 12560 | 10745 | 12534 | 14053 | 10475 | 13965 | 14103 | 12860 | 15246 | 89222 | 12626 | 12686 |  |
| # contigs (>= 1000 bp) | 8237 | 6659 | 6464 | 6633 | 7394 | 7237 | 7439 | 7332 | 6728 | 7772 | 13751 | 6648 | 6701 |  |
| # contigs (>= 5000 bp) | 2082 | 1809 | 1750 | 1808 | 1804 | 1734 | 1869 | 1737 | 1807 | 2059 | 1616 | 1810 | 1841 |  |
| # contigs (>= 10000 bp) | 1159 | 929 | 877 | 931 | 972 | 928 | 1015 | 917 | 933 | 1185 | 953 | 937 | 957 |  |
| # contigs (>= 25000 bp) | 607 | 502 | 467 | 499 | 562 | 540 | 591 | 533 | 520 | 666 | 363 | 527 | 534 |  |
| # contigs (>= 50000 bp) | 332 | 325 | 315 | 325 | 343 | 333 | 347 | 335 | 334 | 387 | 139 | 332 | 340 |  |
| Total length (>= 0 bp) | 91902517 | 90332549 | 89940073 | 90329257 | 90900625 | 88722133 | 90411923 | 90930559 | 90445811 | 90090112 | 95177404 | 90265738 | 90337409 |  |
| Total length (>= 1000 bp) | 86234738 | 86783596 | 87202973 | 86779520 | 86876464 | 86454194 | 86461442 | 86887407 | 86799821 | 85876540 | 61684855 | 86696831 | 86761841 |  |
| Total length (>= 5000 bp) | 72629342 | 75812989 | 76457347 | 75857902 | 74377509 | 74192343 | 73946691 | 74365564 | 75632225 | 73174036 | 40867678 | 75709681 | 75734079 |  |
| Total length (>= 10000 bp) | 66344814 | 69840006 | 70535155 | 69907411 | 68731986 | 68698071 | 68131302 | 68807262 | 69706902 | 67232810 | 36269665 | 69791781 | 69723057 |  |
| Total length (>= 25000 bp) | 57587607 | 63265699 | 64290853 | 63270829 | 62355611 | 62679098 | 61490632 | 62828792 | 63384571 | 59061880 | 26919539 | 63473661 | 63157996 |  |
| Total length (>= 50000 bp) | 47788738 | 56876287 | 58791768 | 56993774 | 54386581 | 55073452 | 52747602 | 55637715 | 56551157 | 49002621 | 19181862 | 56393780 | 55999346 |  |
| # contigs | 12794 | 10614 | 10164 | 10583 | 11890 | 9970 | 11878 | 11834 | 10757 | 12344 | 40609 | 10551 | 10683 |  |
| Largest contig | 1810358 | 1810358 | 1810358 | 1810358 | 1810358 | 1810358 | 1810358 | 1810358 | 2056764 | 1636012 | 1810912 | 1810358 | 2048098 |  |
| Total length | 89437615 | 89532878 | 89807496 | 89526929 | 90021990 | 88600660 | 89562953 | 90033255 | 89600386 | 89071362 | 79944720 | 89409978 | 89526306 |  |
| Reference length | 104990043 | 104990043 | 104990043 | 104990043 | 104990043 | 104990043 | 104990043 | 104990043 | 104990043 | 104990043 | 104990043 | 104990043 | 104990043 |  |
| GC (%) | 52.27 | 52.26 | 52.3 | 52.26 | 52.31 | 52.36 | 52.27 | 52.31 | 52.26 | 52.28 | 52.8 | 52.26 | 52.26 |  |
| Reference GC (%) | 53.52 | 53.52 | 53.52 | 53.52 | 53.52 | 53.52 | 53.52 | 53.52 | 53.52 | 53.52 | 53.52 | 53.52 | 53.52 |  |
| N50 | 60848 | 104755 | 121173 | 105527 | 89983 | 98454 | 82031 | 97274 | 103112 | 61654 | 5680 | 103107 | 98454 |  |
| NG50 | 37782 | 69299 | 80907 | 70279 | 56248 | 59931 | 51166 | 62763 | 67623 | 41161 | 1605 | 68024 | 62707 |  |
| N75 | 9157 | 14447 | 15463 | 14490 | 12097 | 14050 | 11492 | 12220 | 14273 | 10545 | 1083 | 14860 | 14361 |  |
| NG75 | 2752 | 3635 | 3921 | 3639 | 3174 | 3091 | 3067 | 3172 | 3580 | 2816 | 519 | 3580 | 3603 |  |
| L50 | 277 | 163 | 140 | 162 | 202 | 182 | 221 | 184 | 175 | 306 | 1448 | 174 | 181 |  |
| LG50 | 440 | 251 | 216 | 250 | 308 | 287 | 343 | 280 | 266 | 464 | 6337 | 265 | 278 |  |
| L75 | 1236 | 704 | 620 | 700 | 862 | 739 | 926 | 802 | 722 | 1144 | 12090 | 710 | 741 |  |
| LG75 | 3756 | 2497 | 2267 | 2486 | 2903 | 2897 | 3097 | 2839 | 2541 | 3556 | 38248 | 2527 | 2549 |  |
| # misassemblies | 71 | 74 | 63 | 73 | 73 | 67 | 73 | 69 | 83 | 110 | 110 | 71 | 70 |  |
| # misassembled contigs | 69 | 71 | 62 | 70 | 70 | 64 | 70 | 67 | 80 | 89 | 105 | 68 | 67 |  |
| Misassembled contigs length | 4364945 | 5170663 | 3153575 | 5063735 | 4113359 | 4139554 | 3694134 | 3789992 | 6224014 | 3199964 | 2699012 | 4824308 | 3345374 |  |
| # local misassemblies | 48 | 53 | 54 | 42 | 221 | 160 | 110 | 213 | 207 | 550 | 20876 | 60 | 88 |  |
| # unaligned contigs | 108 + 689 part | 101 + 258 part | 86 + 216 part | 100 + 251 part | 104 + 586 part | 50 + 485 part | 107 + 570 part | 105 + 572 part | 101 + 306 part | 132 + 332 part | 2253 + 7328 part | 87 + 252 part | 102 + 281 part |  |
| Unaligned length | 180508 | 157651 | 149770 | 156367 | 171501 | 133435 | 169815 | 168104 | 159000 | 201138 | 3044984 | 155140 | 161708 |  |
| Genome fraction (%) | 84.5 | 84.876 | 85.179 | 84.879 | 85.167 | 83.915 | 84.741 | 85.196 | 84.905 | 84.279 | 68.375 | 84.769 | 84.854 |  |
| Duplication ratio | 1.006 | 1.003 | 1.003 | 1.003 | 1.005 | 1.004 | 1.005 | 1.005 | 1.003 | 1.004 | 1.071 | 1.003 | 1.003 |  |
| # N's per 100 kbp | 0 | 0 | 0 | 0 | 0 | 0 | 0 | 0 | 0 | 0 | 0 | 0 | 0 |  |
| # mismatches per 100 kbp | 22.7 | 19.98 | 17.92 | 19.73 | 21.08 | 18.99 | 19.57 | 20.99 | 21.05 | 19.26 | 18.43 | 20.03 | 20.52 |  |
| # indels per 100 kbp | 1.13 | 0.84 | 0.81 | 0.84 | 1.52 | 1.24 | 1.17 | 1.41 | 1.1 | 1.46 | 47.78 | 0.88 | 1.08 |  |
| # predicted genes (unique) | 90166 | 88941 | 88516 | 88923 | 89816 | 87292 | 89776 | 89766 | 89130 | 89615 | 104535 | 88814 | 88991 |  |
| # predicted genes (>= 0 bp) | 90368 | 89055 | 88978 | 89037 | 90290 | 87785 | 89894 | 90250 | 89250 | 89706 | 104658 | 88927 | 89100 |  |
| # predicted genes (>= 300 bp) | 77183 | 76392 | 76389 | 76386 | 77222 | 75378 | 76873 | 77193 | 76511 | 76554 | 76075 | 76275 | 76420 |  |
| # predicted genes (>= 1500 bp) | 10348 | 10671 | 10793 | 10678 | 10580 | 10555 | 10488 | 10600 | 10627 | 10418 | 5801 | 10653 | 10642 |  |
| # predicted genes (>= 3000 bp) | 958 | 1026 | 1045 | 1027 | 1002 | 1000 | 988 | 1008 | 1027 | 966 | 459 | 1022 | 1024 |  |
| Largest alignment | 1636012 | 1636012 | 1636012 | 1636012 | 1636012 | 1636012 | 1636012 | 1636012 | 1719883 | 1636012 | 1257163 | 1636012 | 1636012 |  |
| Total aligned length | 89227711 | 89362652 | 89647673 | 89358704 | 89818248 | 88440170 | 89363157 | 89840073 | 89423570 | 88853207 | 76179652 | 89241864 | 89348132 |  |
| NA50 | 60007 | 102577 | 119328 | 104441 | 89603 | 98240 | 80672 | 94846 | 101992 | 60937 | 5623 | 102156 | 97403 |  |
| NGA50 | 37434 | 68907 | 80606 | 69264 | 56121 | 58514 | 50782 | 62707 | 67510 | 40157 | 1525 | 66925 | 62395 |  |
| NA75 | 9067 | 14234 | 15320 | 14273 | 11816 | 13924 | 11319 | 11994 | 14003 | 10278 | 992 | 14547 | 14129 |  |
| NGA75 | 2702 | 3581 | 3859 | 3586 | 3118 | 3051 | 3027 | 3121 | 3533 | 2778 | - | 3533 | 3551 |  |
| LA50 | 281 | 168 | 143 | 166 | 207 | 187 | 226 | 188 | 180 | 310 | 1463 | 177 | 185 |  |
| LGA50 | 446 | 257 | 220 | 255 | 314 | 293 | 348 | 284 | 272 | 471 | 6514 | 270 | 282 |  |
| LA75 | 1252 | 718 | 630 | 714 | 876 | 751 | 939 | 814 | 736 | 1166 | 12688 | 722 | 752 |  |
| LGA75 | 3806 | 2539 | 2299 | 2528 | 2951 | 2943 | 3143 | 2884 | 2584 | 3626 | - | 2566 | 2588 |  |
| Total Misassemblies | 119 | 127 | 117 | 115 | 294 | 227 | 183 | 282 | 290 | 660 | 20986 | 131 | 158 |  |
